# A Sustainable Reuse of Agro-Industrial Wastes into Green Cement Bricks

**DOI:** 10.3390/ma15051713

**Published:** 2022-02-24

**Authors:** Wei Quan Chin, Yeong Huei Lee, Mugahed Amran, Roman Fediuk, Nikolai Vatin, Ahmad Beng Hong Kueh, Yee Yong Lee

**Affiliations:** 1 Department of Civil and Construction Engineering, Faculty of Engineering and Science, Curtin University Malaysia, CDT 250, Miri 98009, Sarawak, Malaysia; 700017902@student.curtin.edu.my (W.Q.C.); yhlee@civil.my (Y.H.L.); 2 Department of Civil Engineering, College of Engineering, Prince Sattam Bin Abdulaziz University, Alkharj 16273, Saudi Arabia; 3 Department of Civil Engineering, Faculty of Engineering and IT, Amran University, Amran 9677, Yemen; 4 Polytechnic Institute, Far Eastern Federal University, 690922 Vladivostok, Russia; fedyuk.rs@dvfu.ru; 5 Peter the Great St. Petersburg Polytechnic University, 195251 St. Petersburg, Russia; vatin@mail.ru; 6 Department of Civil Engineering, Faculty of Engineering, Universiti Malaysia Sarawak, Kota Samarahan 94300, Sarawak, Malaysia; kbhahmad@unimas.my (A.B.H.K.); yylee@unimas.my (Y.Y.L.)

**Keywords:** agro-industrial waste, POFA, oil palm shell, quarry dust, calcium carbonate, brick

## Abstract

The fabrication of bricks commonly consumes relatively high natural resources. To reduce the carbon footprint in the brick production industry, repurposing industrial wastes in the making of sustainable bricks is a recent trend in research and application. Local wastes, such as oil palm shell (OPS), palm oil fuel ash (POFA), and quarry dust (QD), are massively produced annually in the palm oil-exporting countries. Moreover, QD from mining industries is hazardous to both water and air quality. For better waste management in marching towards sustainability, these wastes should be given their second life as construction materials. Therefore, this paper investigates the possibility of incorporating agro-industrial wastes into the brick mixture by examining their properties by means of several standardized tests. For the mix design, a 100% replacement of coarse aggregate with OPS, 20% replacement of cement with POFA, 20% cement weight of limestone as admixture, and 0 to 50% replacements of fine aggregate with QD are experimentally considered. The optimum mix of these wastes is preliminarily determined by focusing on high compressive strength as an indicator. Other examinations include splitting tensile, flexural strength, water absorption, and efflorescence tests. Although the agro-industrial waste cement brick is 18% lower in the strength to weight ratio compared to that of conventional, it is observed that it has better late strength development due to its POFA pozzolanic properties. Moreover, the proposed green cement brick is further checked for compliance with several standards for feasible use in the construction industry. Financially, the cost for the brick with the new mix design is almost equivalent to that of conventional. Hence, this green cement brick is reasonable to be employed in the construction industry to promote material sustainability for better waste management.

## 1. Introduction

Bricks are common building materials actively applied in the vast majority of construction works. Because of their remarkable features, such as high strength, low production costs, and durability, bricks have played an important role in construction for thousands of years [1,2,3]. Brick was an important building material in Egypt, Roman, and Mesopotamia [4]. As cement was introduced for concrete binder, clay brick was also innovatively developed into cement brick to reduce the consumption of heating energy that is used to produce clay bricks. Both clay and cement bricks are used in the current construction industry. Improving sustainability is one of the most difficult tasks confronting the construction industry, and alternative building materials are being explored to offset environmental impacts and meet standards for sustainable development, manufacturing, and consumption [5,6]. Sustainability supports multifaceted and interdisciplinary collaboration in the building sector to meet human needs, including the requirement for housing, and to organize quality settings for society [7]. Production of bricks is an energy-taxing process that consumes considerable natural resources, namely limestone, clay, gravel, river sand, etc. [8]. In order to remedy this, sustainable materials are in recent years beginning to be introduced as alternatives to conventional brick materials to reduce carbon footprint. Agriculture wastes such as rice husk ash [9,10] and wastewater sludge [11], tannery sludge [12], dust waste [13], etc., are merely some of the numerous materials now considered for this purpose. As one of the largest palm oil-producing countries, Malaysia is found to generate massive wastes in terms of dry mass resulting from the production activity [14], from which great remedial measures must be exercised. Particularly, the identified wastes from the palm oil industry are oil palm shell (OPS) and palm oil fuel ash (POFA), where they can be repurposed as aggregate and cement replacements, respectively, to produce sustainable bricks in matching with the global efforts in improving existing waste management.

The building and demolition industry accounts for 40% of global energy consumption and one-third of greenhouse gas emissions [15,16]. According to reports, as the need for building materials increased, so did the use of raw materials and energy, particularly during the extraction, processing, and material transit phases [17]. The current worldwide volume of solid waste creation is about 2.01 billion tons per year, and it is anticipated to rise to about 3.40 billion tons per year by 2050 [18]. The use of solid industrial waste as substitute stabilizers in building activities has proven to be a viable strategy for decreasing environmental effects while also providing social and economic benefits [19,20]. The worldwide urban growth is one of the primary causes of the significant increase in solid waste output. Emerging nations choose irregular landfill removal and open dumps [21,22]. As a result, it is said, for example, that recycling wastes by incorporating them into construction materials offers a remedy to these concerns concerning the disposal of waste and ecological impact reduction [23]. According to their research, using 1% cigarette butts into sintered clay bricks can save roughly 10.2% of the burning energy used in the production process [23].

Because of its mechanical and physical characteristics, as well as the creativity of combining diverse wastes in its manufacturing, the masonry block is one of the most comprehensive building material components [23]. Despite its outstanding workability and availability, it is well known that the manufacturing of sintered masonry blocks has been a very intensive operation in terms of resources and energy, in addition to the significant amounts of carbon consumed [2,24,25,26]. According to reports, reducing energy usage is a primary emphasis of civil construction [27]. The increased demand for sustainable and environmentally friendly products has prompted research into alternate methods and materials for producing building bricks [6,28]. It has also been discovered that the raw materials consumed by the building sector account for an estimated 24% of the world’s raw material supply [29]. Thus, in order to fulfill the maintainable development goal, the choice of construction material is critical. Soil-cement blocks offer a solution to these problems since they are simply created using a procedure that eliminates the need for burning, reduces the amount of cement used, and still allows for the use of waste materials in their composition. Furthermore, the use of cement blocks decreases expenses by up to 40% when compared to traditional masonry, particularly in low-cost housing [7]. As a result, cement blocks might be deemed more environmentally friendly than typical masonry blocks [6,30].

Natural resource shortages and the formation of solid waste without effective disposal are global concerns [25,31,32,33,34], and this permits ecological viability for construction systems, encouraging ecological sustainability and process optimization [35,36]. The current global concerns stem from extensive ecological concerns, as well as the expedited rate of technology innovation in the industry, particularly in construction, and, as a result, attention to the concept of alternative building materials, particularly materials produced of the earth, has grown [37]. Because of their poor thermal conductivity, stabilized soil blocks aid increase construction energy efficiency. As a result, they can be used to improve acoustic and thermal insulation in buildings [38]. However, in terms of engineering properties, the abrasion value for OPS is 4.8%, which is the amount of the original oil palm kernel samples ground to smaller than 1.7 mm diameter, and therefore less likely to deteriorate when used as a base fill [39]. Oil palm shells also have small pores between their fibers that further provide their lightweight characteristic without degradation in strength [40,41,42,43,44]. To date, massive research has been conducted on structural OPS concretes [45,46,47,48,49,50], where the inclusion of palm oil industry wastes has shown excellent overall concrete properties and durability while incorporating extra advantages of load-carrying characteristics despite being low mass. Moreover, quarry dust (QD) from the mining industry has been identified as useful material for the concrete matrix, attributing to some research on its potential as an aggregate replacement [51,52,53,54,55,56,57]. So, QD can be reused in concrete as a building material to reduce the by-product waste disposal into the surrounding environment since severe health issues can be one of the consequences [58].

There is a high demand for cement bricks in the current construction industry, and Malaysia produces tons of waste from industries. In order to incorporate all these local wastes into the concrete matrix and better waste management, this paper investigates, therefore, the design mix feasible to be applied in this proposed green cement bricks towards achieving the aim of manufacturing sustainable construction materials. The main focus is to utilize local all wastes to fully replace coarse aggregate in cement brick which is rarely found in current research trends. To obtain the mechanical properties of the proposed mixes, the experimental program for this study includes compressive, splitting tensile, flexural strengths, water absorption, and efflorescence tests. Additionally, their feasibility as sustainable construction materials is identified through appraisal of compliance with the current local codes. A cost analysis has also been considered comparable to that of conventional to seek its production financial impact. The outcomes should benefit and promote sustainability in the construction industry.

## 2. Experimental Investigation

The current experimental program for studying the feasibility of local wastes for brick production was carried out in three phases. The mixture was designed and identified in Phase 1, properties characterization was performed in Phase 2, while Phase 3 was devoted to the code compliance and cost analysis.

### 2.1. Materials

The identified wastes were POFA, OPS, and QD, where they were, respectively, functioned as the cement, coarse aggregate, and fine aggregate replacements to produce a lightweight concrete mix for brick production.

**Figure 1:**
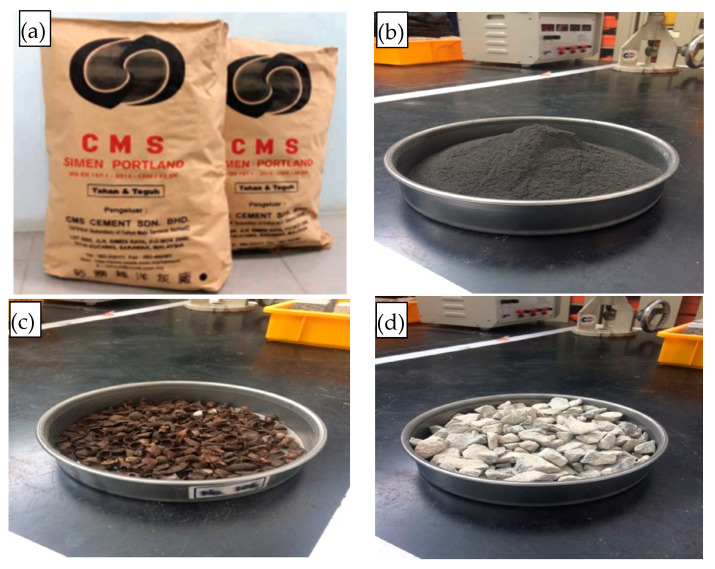
Materials used in the design mix, (**a**) OPC, (**b**) POFA, (**c**) OPS and (**d**) gravels.

Additional details for the investigated materials are: Cement—Ordinary Portland cement (OPC), CMC Engineering Sdn Bhd, Selangor, Malaysia; a local product that complied with MS EN 197-1 and ASTM C150; POFA (Curtin University Lab, Sarawak, Malaysia) was considered as the supplementary cementitious material with sizes passing through 45 μm sieve and heat-treated at 500 °C; OPS (Malaysian palm oil industry, Sarawak, Malaysia) complied with ASTM C33 for the sizing; QD (quarry dust, Sarawak Malaysia) with the density of 1350 kg/m^3^ and sieved to maintain its fineness modulus range of 2.3 to 3.1 (previous properties also have been included in Table 1 as a reference); River sand (Sarawak valley, Malaysia) with the density of 1420 kg/m^3^ and passing 4.75 mm sieve size; superplasticizer of BASF-Master Glenium ACE 8589 type; (MasterGlenium—Master Builders Solutions, Sarawak, Malaysia) and calcium carbonate powder (Supra-coat WCE-22, Shandong Alpa Powder Technology Co., Ltd., Shandong, China) as the admixture to promote early strength to the overall concrete matrix. Figure 1 shows the materials of the design mix.

#### Particle Size Distribution

**Figure 2:**
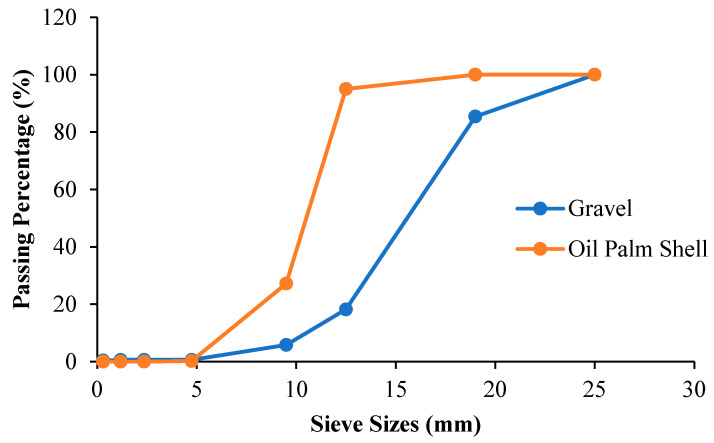
Comparison of the coarse aggregate grading of gravel and OPS.

The main purpose of the sieve analysis was to determine the particle size distribution for each material used to cast the concrete in complying with the ASTM C330M [62] and C33M [63] standards. Several fine aggregate mixtures were proposed and tabulated in Table 2, where only the mix with 50% of QD and 50% of river sand grading followed the ASTM C33M requirement. The coarse aggregate grading is shown in Figure 2 for gravel and OPS. Both aggregates can pass through the nominal diametric sieve size of 4.75 mm.

### 2.2. Mix Design

The mix design was fixed at 1:1:1 for cement, fine, and coarse aggregates according to [39]. In Phase 1, an identical mix of binder (80% OPC + 20% POFA), coarse aggregate (100% OPS), and fine aggregate (50% QD + 50% RS) were considered with the water-cement ratio and superplasticizer content as variables. From the preliminary study, both water-cement ratios of 0.45 and 0.5 were acceptable, while a 1% cement weight of the superplasticizer was determined as meeting the program objective in this stage. The mixes were able to flow with total slump, and the compressive strength exceeded 17 MPa of structural use. The equivalent weight of limestone powder with POFA was added to the mixture to activate the reaction of POFA. In Phase 2, 10%, 30%, and 50% QD were examined along with the consideration of a control case of conventional concrete specimen for comparison. Table 3 shows the preliminary design mix in Phase 1. Table 4 shows the mix design of Phase 2.

### 2.3. Methods of Testing

In the following, the conducted tests for the characterization of the concrete specimens are described. All specimens were tested according to test procedures in codes of practice. Only one specimen was tested for slump, density, efflorescence, and water absorption tests. However, average values of three specimens were obtained from the strength tests.

#### 2.3.1. Slump Test and Density

Slump tests were carried out in accordance with ASTM C143 [64]. Three layers of the mix were poured to fill up the cone alongside the application of 25 times compaction with the rod tamps for each layer. The cone was then removed without lateral and torsional movements such that the slump height was measured immediately after the removal of the cone. Additionally, the fresh density was measured by weighing the specimen in a cube after 15 min of compaction on three layers of mix according to ASTM C172 [65]. The dry density was determined by weighing the specimen at concrete testing age of 28 days.

#### 2.3.2. Strength Test

**Figure 3:**
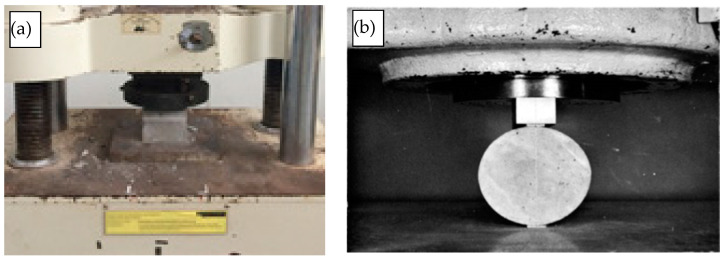

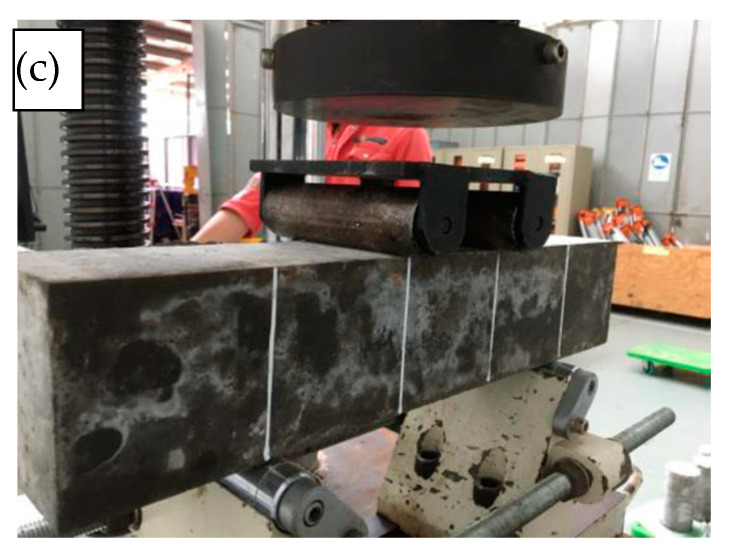
(**a**) compression (**b**) splitting tensile and (**c**) flexural tests setup.

Compressive strength was determined according to ASTM C140 [66] at 7, 14, 21, and 28 days of concrete ages for specimens prepared with a 100 mm cube, as shown in Figure 3. Three specimens were prepared for each of the concrete ages with a loading rate of 0.4 MPa/min. For concrete tension strength, the splitting tensile test was carried out according to the code specifications of ASTM C496 [67]. For this, three cylindrical specimens for each of the concrete ages, with dimensions of 200 mm height and 100 mm diameter, were tested under a loading rate of 1.0 MPa/min. Furthermore, a flexural strength test was conducted for all mixes based on ASTM C78 [68] at 28 days of concrete age using three prepared specimens.

#### 2.3.3. Efflorescence and Water Absorption Tests

**Figure 4:**
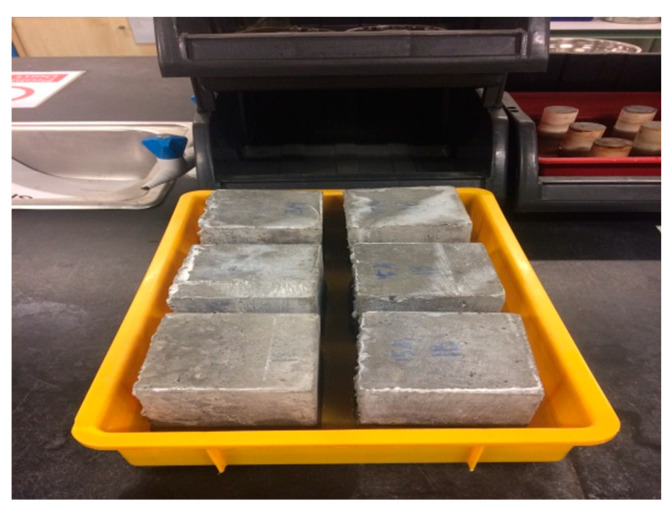
Immersion for efflorescence test.

At the concrete curing age of 28 days, efflorescence and water absorption tests were carried out according to the ASTM specifications [66,69]. Only one specimen was prepared for concrete curing ages of 7 and 28 days. The bricks (with size of 215 × 100 × 65 mm) were placed on their header faces towards the ground in distilled water for seven days after the end of the curing session of 28 days, as shown in Figure 4. The bricks were next heated after the 7-days immersion at 110 °C for 24 h. Then, they were placed at 3 m away from the observer for the examination of any sign of efflorescence at any face of the brick. Any white residue in the distilled water tank was also observed, i.e., looking for any sign of efflorescence or precipitation of mineral salt within the concrete matrix. For the water absorption test, the specimens were oven-dried for 24 h at 110 °C and then immersed in water. Both dry and wet weights were measured to quantify the water absorbance of the specimens.

## 3. Results and Discussions

From Phase 1, the optimum superplasticizer was determined at 1% of the cement weight with a water-cement ratio of 0.45. This was then applied in Phase 2 investigation for various fine aggregate replacements with different proportions of QD. The properties of the design mixes in Phase 2 are discussed in detail in the following sections.

### 3.1. Densities and Slump Properties

From Table 5, the densities of 100% RS are found to be 25.7% and 29.6% lighter than the conventional concrete mixes for fresh and dry densities, respectively. The differences are due to the POFA content replacement of 20% of cement weight. The various replacements with QD show no significance in the fresh or oven-dry densities, with only a slight decrease in densities for higher replacement contents. Compared to conventional concrete, the computed highest reduction in the total dead load coming from the construction material is 35.0%. This will eventually help to reduce the transportation cost of material and also fulfilling the supporting members’ dimension needs. In the context of performance, almost all mixes achieve more than 17 MPa at 28-days of the characteristic concrete strength. The workability of all specimens is considered to have a high slumping value as the range is 209 to 231 mm.

### 3.2. Mechanical Properties

#### 3.2.1. Compressive Strength

**Figure 5:**
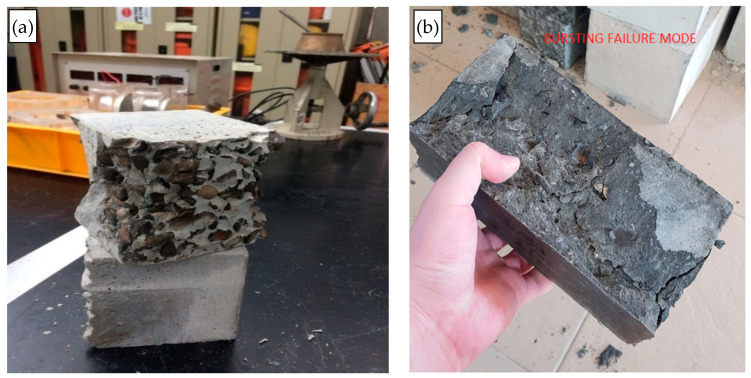
Typical failure modes of the design mixes for (**a**) cube and (**b**) brick specimens.

From Table 6, it is witnessed that the obtained compressive strengths can be categorized as structural concrete according to the ACI code specification of 17 MPa for both cube and brick size specimens. All specimens display a customary increasing trend in strength following the increase in the curing time. Additionally, the corresponding typical failure mode is shown in Figure 5. It can also be noticed in Table 5 that the increment of quarry dust replacement does not cause any effects on the strength gain throughout the curing ages.

From the test observation, due to the low specific gravity of OPS, the design mixes showed tendency to segregate thereby potentially affecting their compressive strength [70]. Compared to previous similar mixes [71], the current finding showed an improvement of strength with the limestone powder inclusion. The filler effects of the limestone powder inflicted the hydration reaction to take place at optimum cement exposure with added water and hence promoting the strength development [72]. Moreover, ettringite was formed when applying limestone in the concrete matrix, which makes the concrete more durable [73].

It can be seen in Table 6 that the growth of compressive strength up to 28-days of concrete age for 30% QD + 70% RS is 29.8%, where it is only 27.5% for the control sample. The incorporation of POFA promotes strength development at a later concrete age with additional pozzolanic reaction because the pozzolanic activity can consume the calcium hydroxide in the interfacial transition zone (ITZ) to improve the binding of concrete with the aggregates. The calcium hydroxide usually resides in the area where concrete and the aggregates meet [74]. Furthermore, the hydroxide compounds commonly need a longer time to form C-S-H and C-A-H gels, which promotes later strength. These reactions concur with the addition of POFA into the concrete matrix due to its mineral composition and pozzolanic properties [40,75]. The SiO_2_ in POFA can react with the Ca(OH)_2_ from cement hydration to produce extra C-S-H bonding [76].

#### 3.2.2. Splitting Tensile Strength

Table 7 summarizes that only 30% QD + 70% RS achieves the structural requirement of 2 MPa specification from the laboratory results. The splitting tensile strength displays the same enhancement trend as that depicted by the compressive strength, where the increment in compressive strength offers a positive effect on the tensile strength. There were several published equations used for the correlation of the splitting tensile based on the compressive strength of the concrete at specified concrete age. These include Equation (1) [39] for OPS as the replacement for coarse aggregate, Equation (2) for conventional concrete mixes [77], Equation (3) for lightweight concrete with densities between 1600 and 1860 kg/m^3^ [78], and Equation (4) for fly ash as cement replacement [79]. Making a comparison with Table 6, Equation (3) is observed to display the most identical result with that of actual as gathered from the laboratory test. From Table 7, Equations (3) and (4) can be used to predict the empirical tensile strength of OPS and expanded clay concrete.

(1) $$f_{t}=0.4887\sqrt{f_{cu}}$$

(2) $$f_{t}=0.2f_{cy}^{0.7}$$

(3) $$f_{t}=0.297\sqrt{f_{cy}}$$

(4) $$f_{t}=0.58\sqrt{f_{cy}}$$

where *f_t_* is the splitting tensile strength, *f_cu_* is the characteristic compressive strength, *f_cy_* is the compressive strength (concrete age is not specified).

#### 3.2.3. Flexural Strength

**Figure 6:**
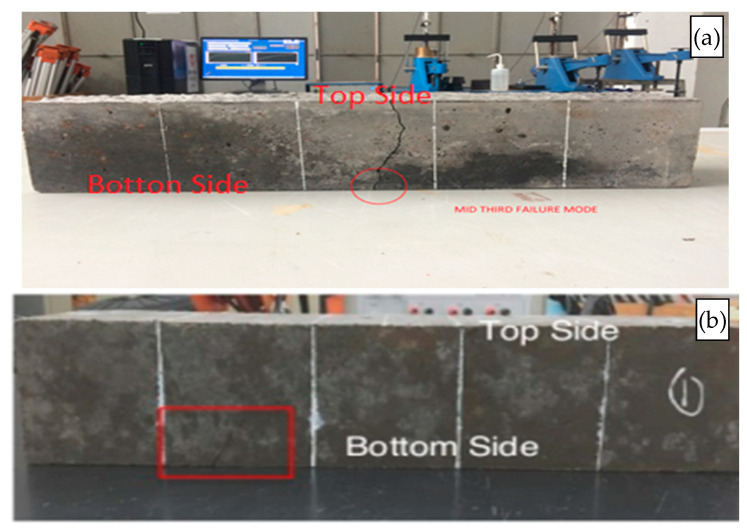
Typical failure of flexural test: (**a**) within central region and (**b**) outside central region.

As summarized in Table 7, the three-point flexural test results also show a similar enhancement trend with those of compressive and tensile strengths. The typical failure can be found in Figure 6, which initiate by cracking. The flexural strength increased when the substitution of sand with quarry dust proportion was increased up to 30%. When the quarry dust reached 50%, the flexural strength tended to perform adversely when compared to those with lesser replacement. This observation matches with that of the previous investigation, which showed that 40% of sand replacement by QD was the optimum design mix [80].

Several equations are readily available to estimate the flexural strength, including Equation (5) for OPS concretes and Equations (6)–(8) for lightweight concretes:

(5) $$f_{r}=0.3\sqrt[{3}]{f_{cu}^{2}}$$

(6) $$f_{r}=0.5225\sqrt{f_{cu}}$$

(7) $$f_{r}=0.46\sqrt[{3}]{f_{cu}^{2}}$$

(8) $$f_{r}=0.58\sqrt{f_{cu}}$$

where *f_r_* is the flexural strength, *f_cu_* is the characteristic compressive strength.

From Table 7, Equations (5), (6) and (8) can be used to predict the empirical flexural strength of OPS and expanded clay concrete. However, Equation (5) demonstrates the most identical prediction values in the cases of the full sand replacement with QD and control specimens. There is not solely one equation that predicts well the flexural strength of all the proposed design mixes. Hence, a further correlation should be established for more accurate strength prediction in the absence of laboratory results.

#### 3.2.4. Water Absorption

**Figure 7:**
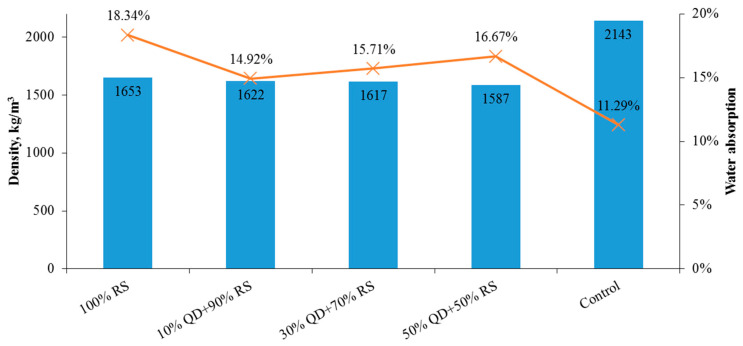
Water absorption of the design mix.

Figure 7 shows that the addition of OPS and more content of QD enhances the water absorption ability of the mixture. OPS may absorb 36% more water than conventional gravel concrete, according to a previous investigation [81]. Lower water absorption was found in the current research as OPS were pre-soaked before mixing with concrete resulting in lower water absorbance tendency.

#### 3.2.5. Efflorescence Test

**Figure 8:**
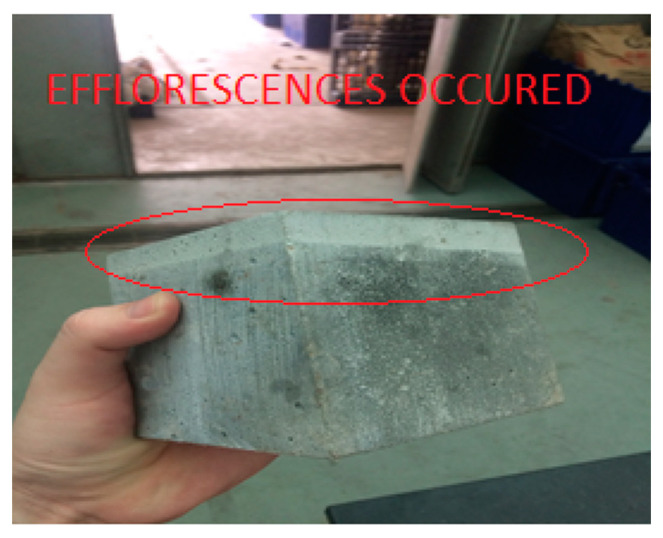
Calcium carbonate deposition layer on the specimen.

All specimens were effloresced where a layer of mineral salt precipitation was noticed on their immersed surfaces, as shown in Figure 8. The primary type of salt formed on the specimens was calcium carbonate, which came from the unreacted Ca(OH)_2_ from the hydration. Ca(OH)_2_ was transported out to the 25 mm distilled water layer as the salt content was lower in the distilled water. After the immersion process, the samples were taken out and dried, during which the precipitation happened as Ca(OH)_2_ was in contact with carbon dioxide to form CaCO_3_.

Specimens with limestone powder may cause extra precipitation due to the reaction of Ca(OH)_2_ in the concrete matrix with the pozzolans (POFA) to form C-S-H gels and thereby increase the density of the concrete while pushing out the excess insoluble CaCO_3_ through the capillaries of the concrete. As the specimens with limestone powder experienced two phases of precipitation, it is justified that the efflorescence visibility was more obvious as compared to the control specimen.

### 3.3. Effects of Drying Shrinkage

Drying shrinkage is one of the most common issues in the concrete industry attributed to its deteriorating effects on concrete strength. Drying shrinkage can be caused by the type of aggregate used, concrete rigidity, water-cement ratio, curing method, environment, etc. According to [82], the normal-weight concrete has a lower drying shrinkage than lightweight concrete, and this is mainly due to the properties and quantities of the aggregate [83]. OPS tends to have a higher water absorption rate than conventional gravel in the concrete matrix [84]. The smooth surface of OPS, with a lower specific surface area as compared to gravel, contributes to the higher drying shrinkage [85]. A lower water-cement ratio may minimize the drying shrinkage, as it has a low possibility of excessive water evaporation [86]. In turn, the minimization of the drying shrinkage may promote the concrete strength.

**Figure 9:**
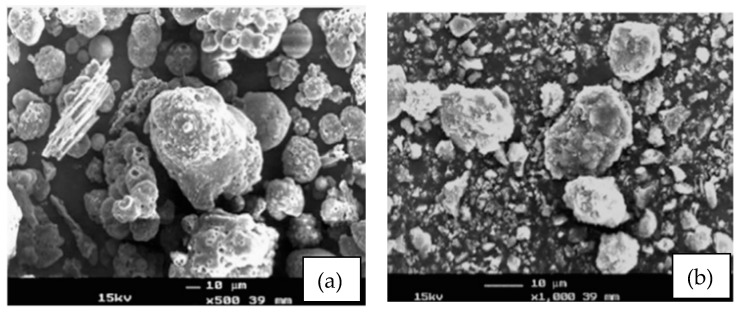
SEM of (**a**) fresh and (**b**) grounded POFA [87].

A higher replacement of quarry dust requires a larger amount of water for the concrete mix to be workable as quarry dust has a high affinity for water, creating a greater moisture suction scenario within the concrete matrix and hence the occurrence of drying shrinkage. This can be solved by prewetting the quarry dust before using it for casting purposes [53]. Furthermore, sand can help to reduce the drying shrinkage due to its filler properties in preventing the formation of water pockets inside the concrete matrix [87]. The magnified illustrations of fresh and grounded POFA are shown in Figure 9. Grounded POFA is able to slip in the gaps between coarse aggregates and undergo pozzolanic reactions [87]. Moreover, these pozzolanic reactions are able to reduce the water pockets in the concrete matrices and hence reduce the drying shrinkage, resulting in a more dense and solid concrete product.

### 3.4. Effects of Saturated Surface Dried Aggregate

Prewetted aggregates or saturated surface dried aggregates are the methods used to saturate the inner pores of the aggregates before casting. The process is executed by soaking the aggregates for 24 h before drying them under the sun or any way appropriate to achieve a dry surface, as suggested by [47]. This process is proposed to prevent the alternation of the designed water content of the mixes. Full hydration may not occur if the water is consumed and attracted by aggregates. Among sun-dried aggregates, air-dried aggregates, and saturated surface dry aggregates, the insertion of saturated surface dried aggregates can produce concrete with the best mechanical properties [88].

### 3.5. Code Specifications

According to Malaysian standards, all mixes considered in the current study do not meet the specifications of engineering blocks. However, they are within the limit of load-bearing brick Classes 1 to 4. For Singapore standards, these mixes comply with the common bricks of Grade 2 and 3 specifications. Generally, the proposed design mixes are only recommended for moderate weather conditions but not severe ones. This is due to the water absorption issue. Water absorption from the surrounding environment is another important performance indicator as this property may also reflect the absorbance of chemicals from the building outdoor to indoor, thus endangering its occupants. In order to address this concern, it is suggested here to apply one impermeable layer of coating to prevent the concrete from absorbing water from the surrounding environment. For a general comparison, Table 8 lists the compliances of the proposed design mixes with numerous existing codes of practice.

### 3.6. Cost Analysis

The preliminary costing of a brick fabrication has been calculated and compared to ordinary OPC brick. Table 9 shows the brick manufacturing cost with the proposed mix of 30% QD + 70% RS. The calculation is based on a single brick with the dimensions: a height of 65 ± 1.875 mm, width of 102.5 ± 1.875 mm, and length 215 ± 3 mm. Overall, the cost to fabricate a single repurposed brick is found cheaper than conventional cement brick. In addition, the beneficial properties for each brick type are included for reference.

Depending on the code specifications, these investigated design mixes serve a wide range of applications for cement brick, as shown in Table 8, from non-load-bearing to load-bearing. By using local industrial wastes incorporated in the brick matrix, they are promoting a sustainable context in the construction industry. Although this green cement brick has lower strength, it is able to achieve structural use according to code specifications. For the high water absorption properties of this proposed green cement brick, it can be solved by introducing the waterproof cover on the concrete surface. As a low-density cement brick, it is also reducing the energy consumption through low heat transfer from the outdoor to the indoor environment [30,89] with lower manufacturing cost, and it should have a good soundproof property due to its low concrete density.

## 4. Conclusions

This study was devoted to the mechanical and physical properties examination of concrete mix with the full replacement of coarse aggregate with OPS, 20% of cement replacement with POFA, and different replacements of fine aggregate with QD as design mixes. Several conclusions can be drawn from the study:

− All design mixes in the second phase met the minimal structural compressive strength of 17 MPa, whereas only 30% QD + 70% RS achieved the minimal tensile splitting strength of 2 MPa for a structural requirement. The compressive strength has been improved as compared to those in [29].
− The water absorptions of all specimens were between 14.9 to 18.3%, with densities of lesser than 1700 kg/m^3^. All design mixes were effloresced where a layer of mineral salt precipitation was found within the specimens.
− It is found that the grounded POFA is able to slip in the gaps between coarse aggregates and undergo pozzolanic reactions. Moreover, these pozzolanic reactions are able to reduce the water pockets in the concrete matrices and hence reduce the drying shrinkage, resulting in a more dense and solid concrete product.
− All design mixes complied with Malaysian (load-bearing brick of Classes 1 to 4), Singapore (common brick of Grades 2 and 3), and ASTM (building and facing bricks at moderate weather conditions) specifications.
− It is revealed that the specimens with limestone powder have experienced two phases of precipitation. This justified that the efflorescence visibility was more obvious as compared to the control specimen.
− The cost to fabricate a single brick was found almost equivalent to a conventional cement brick.

Hence, the proposed green cement brick shows good feasibility to be applied in the construction industry in promoting materials sustainability and better waste management. It is also recommended to conduct a more comprehensive testing program to assess the potential use of the new bricks, such as length change, sound, and thermal insulation properties.

## Figures and Tables

**Table 1:** Physical and chemical properties of QD from previous investigations.

Properties		[59]	[60,61]
Physical	Specific gravity	1.74	2.54–2.60
	Natural water content	6.59%	-
	Water absorption	2.34%	1.2–1.5%
	Bulk density	1.55 kg/m^3^	1720–1810 kg/m^3^
Chemical	SiO_2_	-	62.48%
	Al_2_O_3_	-	18.72
	Fe_2_O_3_	-	6.54
	CaO	-	4.83
	MgO	-	2.65
	Na_2_O	-	-
	K_2_O	-	3.18
	TiO_2_	-	1.21
	Loss of ignition *	-	0.48

* Loss of ignition is the weight loss of a sample after thermal treatment (heating/firing) at high temperature, normally 1000 °C, and 1 h of soaking time.

**Table 2:** Fineness modulus and classification of fine aggregate.

Mixing Proportion	Fineness Modulus	Classification	Compliance to ASTM C33M Grading System
100% RS	0.993	Fine Sand	Not complied
100% QD	3.741	Coarse Sand	Not complied
10% QD + 90% RS	1.312	Fine Sand	Not complied
30% QD + 70% RS	1.612	Fine Sand	Not complied
50% QD + 50% RS	2.562	Well Graded Sand	Complied

RS = river sand, QD = quarry dust.

**Table 3:** Summary of mix design (1 cement: 1 coarse aggregate: 1 fine aggregate) and proportion distribution for Phase 1.

Sample	Binder		Coarse Aggregate		Fine Aggregate		Admixture		Water Cement Ratio
	Cement (%)	POFA (%)	Gravel (%)	OPS (%)	Sand (%)	QD (%)	SP (%)	Limestone (% of Cement)	
1	80	20	0	100	50	50	0.5	20	0.45
2	80	20	0	100	50	50	0.5	20	0.5
3	80	20	0	100	50	50	1.0	20	0.45
4	80	20	0	100	50	50	1.0	20	0.5

**Table 4:** Summary of mix design and proportion distribution for Phase 2.

Specimen	Binder		Coarse Aggregate		Fine Aggregate		Admixture	
	Cement (%)	POFA (%)	Gravel (%)	OPS (%)	Sand (%)	QD (%)	SP (%)	Limestone (% of Cement)
Control	100	0	100	0	100	0	0	0
100% RS	80	20	0	100	100	0	1	20
10% QD + 90% RS	80	20	0	100	90	10	1	20
30% QD + 70% RS	80	20	0	100	70	30	1	20
50% QD + 50% RS	80	20	0	100	50	50	1	20

**Table 5:** Slump results and density measurements for the design mix.

Concrete Sample	Fresh Density, kg/m^3^	Slump (mm)	Slump Characteristic	Oven-Dried Density, kg/m^3^	Performance Index, MPa in a Unit Density
100% RS	1864	231	Total Slump	1653	20.99
10% QD + 90% RS	1840	228	Total Slump	1622	21.58
30% QD + 70% RS	1837	217	Total Slump	1617	22.11
50% QD + 50% RS	1821	209	Total Slump	1587	17.33
Control	2343	107	True Slump	2143	25.28

**Table 6:** Strength at different concrete ages.

Specimen	Compressive Strength (MPa)							
	Cube				Brick			
	7 Day	14 Day	21 Day	28 Day	7 Day	14 Day	21 Day	28 Day
100% RS	22.25	29.13	33.83	34.70	21.80	31.10	34.15	34.76
10% QD + 90% RS	22.25	26.42	33.00	35.00	26.22	38.41	39.63	43.60
30% QD + 70% RS	25.20	29.33	33.33	35.75	25.13	26.83	29.88	47.56
50% QD + 50% RS	21.54	22.71	25.17	27.50	28.66	33.54	38.72	45.73
Control	39.24	40.45	44.55	54.17	56.71	57.32	76.83	77.44

**Table 7:** Splitting tensile strength and flexural strength for concrete specimens with different curing periods.

Sample	Splitting Tensile Strength (MPa)					Flexural Strength (MPa)				
	Actual	Equation (1)	Equation (2)	Equation (3)	Equation (4)	Actual	Equation (5)	Equation (6)	Equation (7)	Equation (8)
100% RS	1.814	2.881	2.397	1.751	1.419	3.236	3.195	3.080	4.899	3.419
10% QD + 90% RS	1.979	3.227	2.810	1.961	2.830	3.512	3.716	3.450	5.698	3.830
30% QD + 70% RS	2.067	3.370	2.986	2.048	3.000	4.322	3.938	3.603	6.038	4.000
50% QD + 50% RS	1.895	3.305	2.905	2.008	2.922	2.761	3.836	3.533	5.883	3.922
Control	3.423	4.301	4.201	2.613	4.104	5.501	5.450	4.598	8.357	5.104
Two-tailed *p* value Paired *t* test at 95% confidence level	-	0.0002	0.0003	0.3895	0.0757	-	0.5510	0.5077	0.0014	0.5373
t	-	12.5696	11.4369	0.9643	2.3833	-	0.6502	0.7266	7.8173	0.6740
*df*	-	4	4	4	4	-	4	4	4	4
Standard error of difference	-	0.094	0.072	0.165	0.260	-	0.247	0.294	0.295	0.280
difference	-	statistically significant	statistically significant	not statistically significant	not statistically significant	-	not statistically significant	not statistically significant	statistically significant	not statistically significant

**Table 8:** Code compliance for the design mix.

Spec.	Code	Class	Compressive Stress (MPa)	Water Absorption (%)	Compliance			
					100% RS	10% QD + 90% RS	30% QD + 70% RS	50% QD + 50% RS
Engineering Block	Malaysian Standards 7.6:1972	A	69.0	4.5	×	×	×	×
		B	48.5	7.0	×	×	×	×
Load Bearing Brick	Malaysian Standards 7.6:1972	15	103.50	-	×	×	×	×
		10	69.0	-	×	×	×	×
		7	48.5	-	×	×	×	×
		5	34.5	-	√	√	√	×
		4	27.5	-	√	√	√	√
		3	20.5	-	√	√	√	√
		2	14.0	-	√	√	√	√
		1	7.0	-	√	√	√	√
Damp Proof Brick	Malaysian Standards 7.6:1972	DPC	7.0	4.5	×	×	×	×
Facing/Common Brick	Singapore Standards 103:1974	1st Grade	35.0	25.0	×	√	√	×
		2nd Grade	20.0	25.0	√	√	√	√
		3rd Grade	5.2	25.0	√	√	√	√
Building Brick	ASTM C62M	SW	20.7	17.0	×	√	√	√
		MW	17.2	22.0	√	√	√	√
Facing Brick	ASTM C216M	SW	20.7	17.0	×	√	√	√
		MW	17.2	22.0	√	√	√	√
Pedestrian Traffic Paving Brick	ASTM C902M	SW	55.2	8.0	×	×	×	×
		MW	20.7	14.0	×	×	×	×
Load Bearing Masonry	ASTM C90M	SW	20.7	17.0	×	√	√	√
		MW	13.1	17.0	×	√	√	√

SW—Severe Weather, MW—Moderate Weather, ×—no and √—yes.

**Table 9:** Cost calculation of a single proposed brick.

OPC brick	Material	Price (USD)	**Benefit**
	Cement	0.21	Stronger than agro-industrial waste incorporated brick Less preparation time Materials highly available
	Gravel	0.01	
	Sand	0.01	
	Water	0.00	
	Labor Cost	0.05	
	Total	0.28	
Proposed agro-industrial waste brick	Cement	0.09	Less cement powder is required for the mix as it is partially replaced by POFA Materials used are mostly sustainable such as incorporating agro-industrial waste into the mix Lower carbon footprint in producing the concrete brick Lighter in weight making it convenient for transportation
	POFA	Waste	
	Sand	0.01	
	QD	0.00	
	Superplasticizer	0.01	
	Water	0.00	
	Labor Cost	0.05	
	OPS	Waste	
	Total	0.16	

## Data Availability

Data sharing not applicable.
